# Intensity‐modulated radiation therapy for multiple targets with tomotherapy using multiple sets of static ports from different angles

**DOI:** 10.1002/acm2.12874

**Published:** 2020-04-06

**Authors:** Yoshihiko Manabe, Yuta Shibamoto, Taro Murai, Akira Torii, Masanari Niwa, Takuhito Kondo, Dai Okazaki, Chikao Sugie

**Affiliations:** ^1^ Department of Radiology Nagoya City University Graduate School of Medical Sciences Nagoya Japan

**Keywords:** dynamic‐couch and dynamic‐jaw mode, intensity‐modulated radiation therapy, multiple targets, static‐port tomotherapy, tomotherapy

## Abstract

**Background:**

To treat multiple targets separated in the craniocaudal direction within a short time, we invented a new technique using multiple static‐port tomotherapy with the dynamic‐jaw mode and named it the pseudo‐DJDC (pDJDC) technique. We compared the pDJDC plans and helical tomotherapy plans using the dynamic‐jaw mode (HDJ) for multiple targets. In the pDJDC plans, we used a beam set with 2–7 ports to the targets at the same level in the craniocaudal direction, and employed another beam set for other targets using different port angles (9–12 angles in total).

**Methods:**

In seven patients, two plans using the pDJDC and HDJ techniques were compared. For multiple targets (n = 2–6), 20–60 Gy in 2‐ to 7.5‐Gy fractions were prescribed for the planning target volumes at D50%. The conformity index, uniformity index (D5%/D95%), dose distribution in the lung, and treatment time were evaluated.

**Results:**

The median conformity index of all seven patients was 3.0 for the pDJDC plans and 2.4 for the HDJ plans (*P* = 0.031). The median uniformity indices of the planning target volume (n = 25) for the two plans were 1.048 and 1.057, respectively (*P* = 0.10). For five patients with thoracic targets, the median mean lung doses were 2.6 Gy and 2.4 Gy, respectively (*P* = 0.63). The median V5Gy and V20Gy of the lungs in the five patients were 11.8% and 8.5% (*P* = 0.63), and 1.6% and 2.1% (*P* = 0.31), respectively. The pDJDC plans reduced the treatment time by 48% compared to the HDJ plans (median: 462 and 884 sec, respectively, *P* = 0.031).

**Conclusion:**

The pDJDC technique allows treatment of multiple targets in almost half the time of the HDJ technique. The pDJDC plans were comparable to the HDJ plans in dose distribution, although the conformity index deteriorated.

**Abbreviations**
DJDC: dynamic‐jaw and dynamic‐couch mode
HDJ: helical‐dynamic‐jaw mode
PTV: planning target volume

## 1 INTRODUCTION

The standard treatment for patients with multiple metastatic lesions is systemic chemotherapy. However, the combined use of radiation therapy, to utilize the beneficial immune‐stimulating effect of focal irradiation, seems to be attracting attention.^1^, ^2^ Treating all lesions, if possible, rather than only treating large lesions, is now advocated by some investigators.^1^, ^3^

**Fig. 1:**
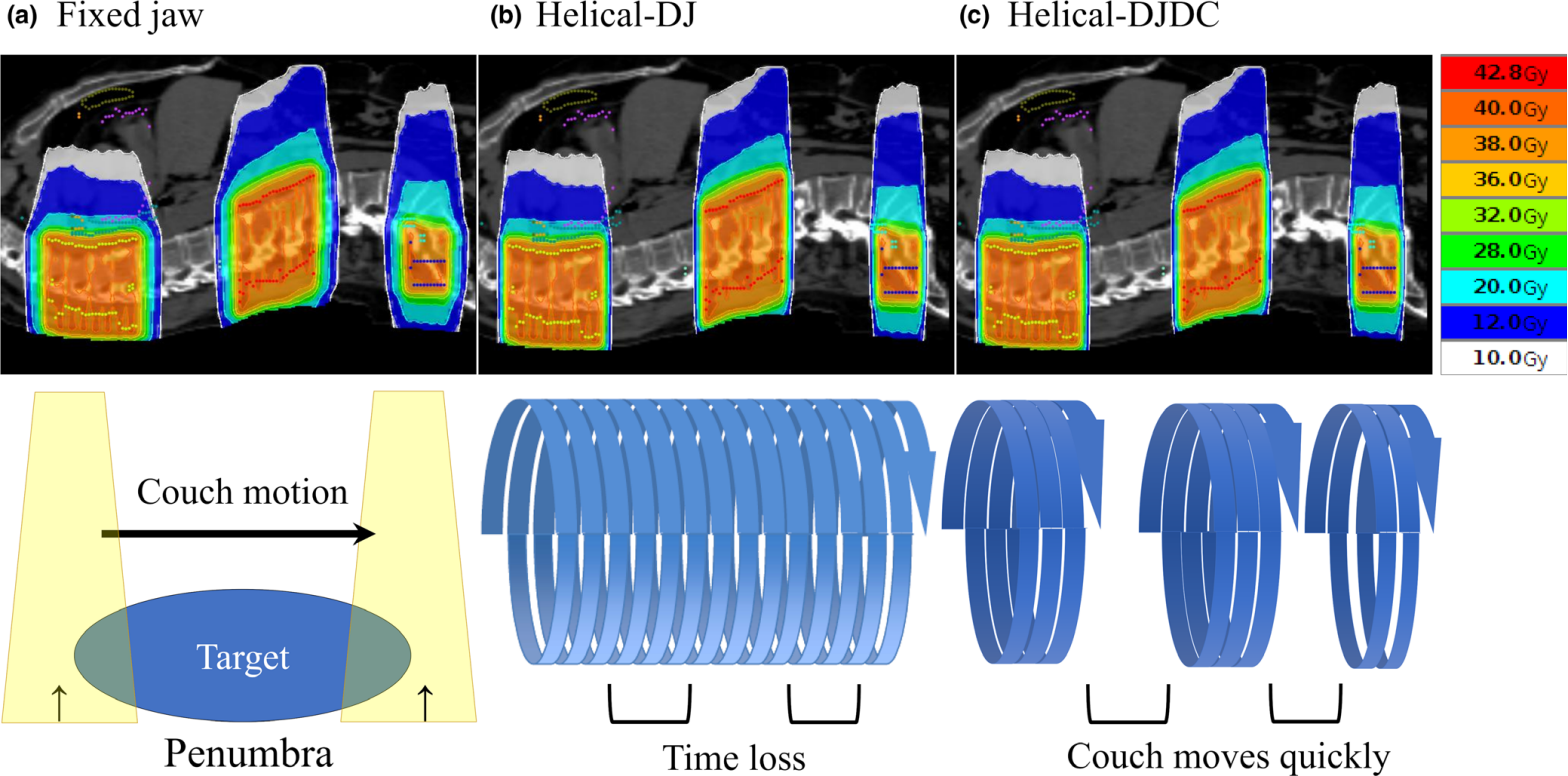
Dose distributions for the treatment of multiple vertebral metastases. (a) Conventional helical fixed‐jaw tomotherapy causes the craniocaudal excess dose. (b) Helical tomotherapy with the dynamic‐jaw mode reduces the craniocaudal excess dose. However, the treatment time becomes prolonged when the targets are far apart in the craniocaudal direction. (c) Helical tomotherapy with the dynamic‐jaw and dynamic‐couch mode reduces the treatment time by allowing faster movement over unirradiated parts of the body.

TomoTherapy® (Accuray Inc., Sunnyvale, CA, USA) is a radiation delivery system that combines dynamic intensity‐modulated radiation therapy and an on‐board imaging system using megavoltage computed tomography.^4^, ^5^, ^6^, ^7^, ^8^, ^9^ Originally, tomotherapy was designed for helical beam delivery, but thereafter a treatment mode using multiple static ports was developed and installed; this treatment was called “topotherapy.”^8^ To treat multiple targets at once, tomotherapy is suitable because the couch can move longitudinally during irradiation of the targets. The maximum distance between the cranial and caudal edges of the targets to be treated at once is 135 cm.^9^ Conventional tomotherapy only had a fixed‐jaw mode and this caused the craniocaudal “penumbra,” that is, excess dose due to thick field width at the craniocaudal edges of a target [Fig. 1(a)]. Recently, the dynamic‐jaw mode was developed [Fig. 1(b)]. This mode sharpens the dose distribution in the craniocaudal edge of the target. We previously reported the clinical usefulness of tomotherapy with dynamic‐jaw mode.^10^, ^11^, ^12^

**Fig. 2:**
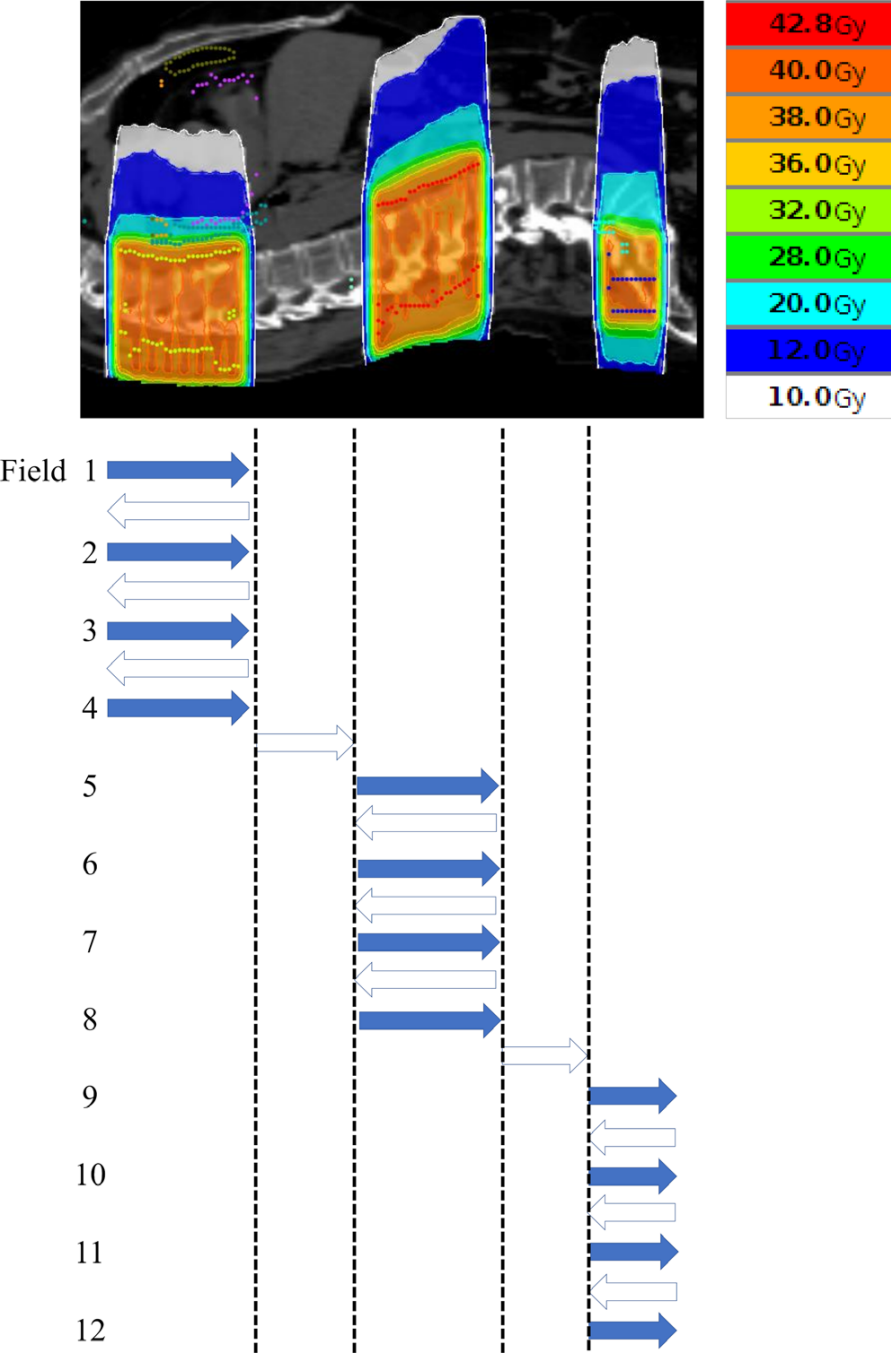
Concept of the pseudo‐dynamic‐jaw and dynamic‐couch technique using multiple static‐port tomotherapy. First, the cranial target is covered by a beam set of fields 1–4. Then, the couch moves quickly and the other targets are covered by fields 5–8 and 9–12. The arrows indicate the couch movement. The couch moves slowly like helical tomotherapy while the beams are on (blue arrows), but it moves quickly when the beams are off (blank arrows).

Helical‐dynamic‐jaw mode (HDJ) allows treatment of multiple targets at once, with a good dose distribution. However, the treatment time becomes prolonged when the targets are far apart in the craniocaudal direction, since the couch moves slowly at a uniform pace even when the gantry is passing unirradiated parts between the targets [Fig. 1(b)]. The dynamic‐jaw and dynamic‐couch (DJDC) mode offers dynamic‐jaw alignment throughout the treatment and a variable couch speed, allowing faster movement over unirradiated parts of the body, but it is not yet available for clinical use [Fig. 1(c)].^13^ To solve the problem of the long treatment time, we invented a new technique using multiple static‐port tomotherapy with the dynamic‐jaw mode, naming it the pseudo‐DJDC (pDJDC) technique (Fig. 2). In this study, we compared the pDJDC plans and HDJ plans for multiple targets, and evaluated the usefulness of the pDJDC technique.

## 2 MATERIALS AND METHODS

### 2.A Study approval and patients

This study was approved by our institutional review board (No. 60‐17‐0021). The study subjects were seven patients (four males and three females) aged 32–82 yr (median, 66). All patients gave written informed consent before entry to the study. In all seven patients, two plans using the pDJDC and HDJ techniques were compared. The patient characteristics are summarized in Table 1.

**Table 1:** Patient characteristics.

Patient	Tumor and location	Target no.	Total PTV (ml)	Port no.^a^	Total dose/ fraction
A	Pleural disseminations from lung cancer	2	33.6	9	60 Gy/8 fr
B	Bone metastases from breast cancer (Rib, Lumbar vertebrae ~ Sacrum, Ilium)	4	943.6	9	40 Gy/20 fr
C	Liver and abdominal wall metastases, Peritoneal dissemination from colon cancer	6	100.0	9	55 Gy/25 fr
D^b^	Tongue, Esophagus, Mediastinal lymph node	3	110.0	12	20 Gy/10 fr
E	Bone metastases (Cervical vertebrae, scapula) from cervical cancer	2	762.0	9	35 Gy/10 fr
F^c^	Esophagus, Mediastinal and supraclavicular lymph node	3	121.5	9	22 Gy/11 fr
G	Bone metastases (Rib and lumbar vertebrae), Inguinal lymph node metastases from lung cancer	5	391.4	12	25 Gy/5fr

Abbreviations: PTV, planning target volume.

^a^ Number of ports for the pseudo‐dynamic‐jaw and dynamic‐couch technique.

^b^ Tongue and esophageal cancer with a regional lymph node metastasis. The pseudo‐dynamic‐jaw and dynamic‐couch technique was used for the boost plan.

^c^ Esophageal cancer with two regional lymph node metastases. The pseudo‐dynamic‐jaw and dynamic‐couch technique was used for the boost plan.

### 2.B The CT simulation and planning

All patients were immobilized in a supine position with a vacuum bag system (BodyFIX; Medical Intelligence, Schwabmünchen, Germany) alongside the whole body. Axial non‐contrast‐enhanced computed tomography with a slice thickness of 2 mm was performed for treatment planning in the supine position under normal breathing. Contouring of target volumes and normal structures was performed on the Pinnacle^3^ version 9 treatment planning system (Philips Medical System, Eindhoven, The Netherlands). The contours created in the treatment planning system were exported to the tomotherapy treatment planning system (Tomo HD version 2.0), where all plans were generated. The clinical target volume was defined as the visible gross tumor volume. We defined the planning target volume (PTV) margin for the clinical target volume as 5 mm in all directions.

**Fig. 3:**
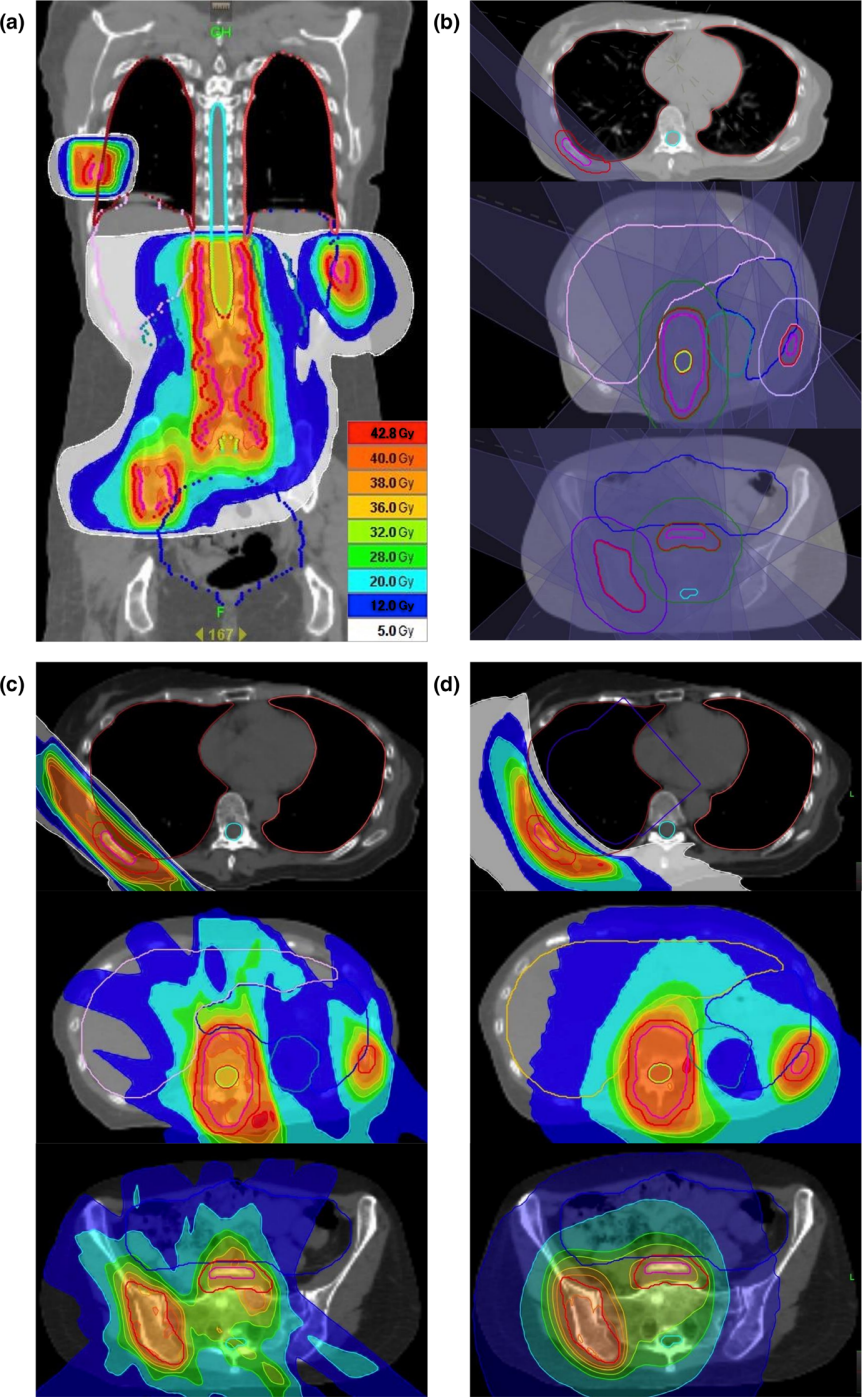
Dose distributions for multiple bone metastases using the pseudo‐dynamic‐jaw and dynamic‐couch technique (pDJDC; a, c) and helical tomotherapy with dynamic‐jaw mode (d). The beam setting for the pDJDC plan is also shown (b).

**Fig. 4:**
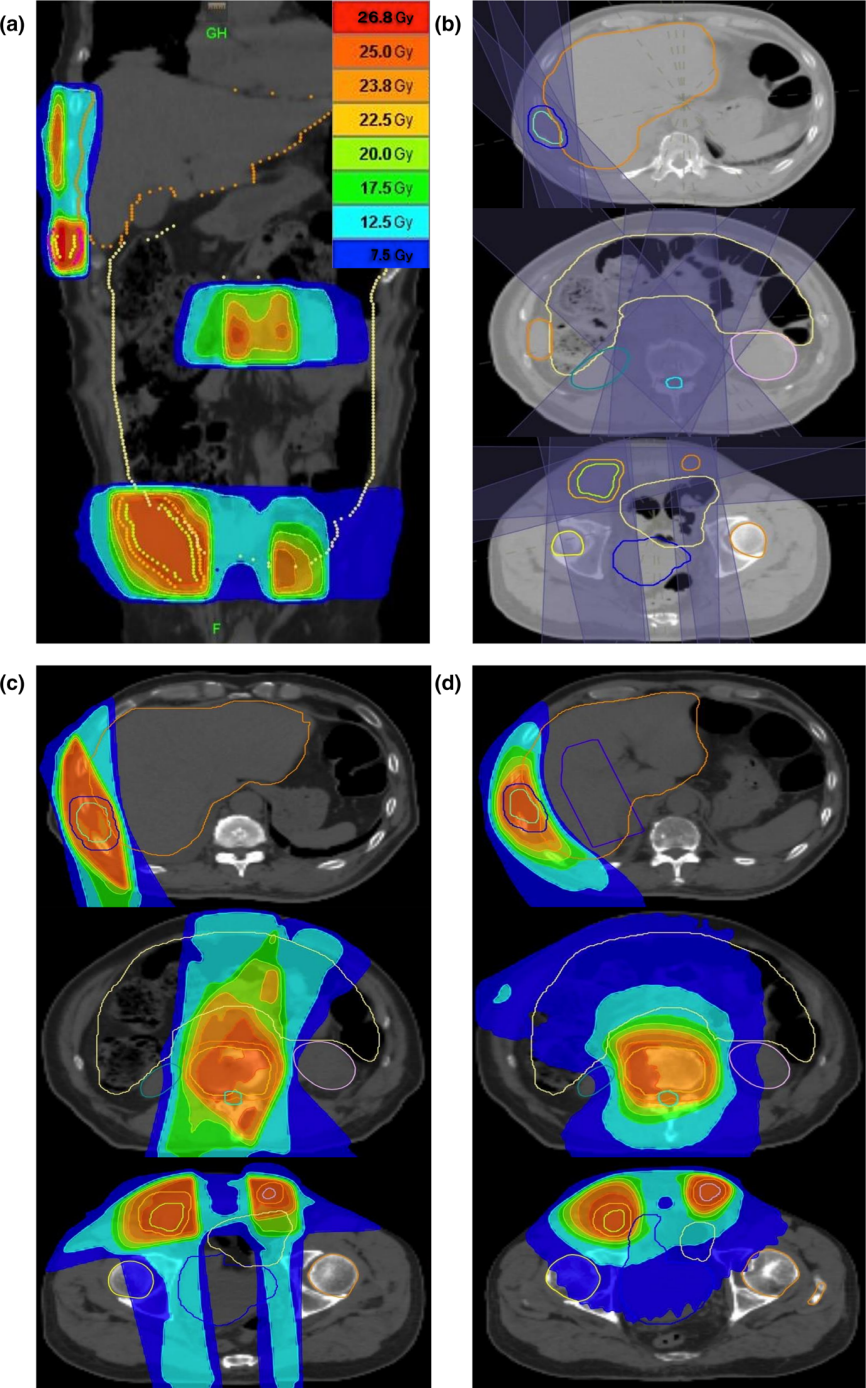
Dose distributions for multiple bone and lymph node metastases using the pseudo‐dynamic‐jaw and dynamic‐couch technique (pDJDC; a, c), and helical tomotherapy with dynamic‐jaw mode (d). The beam setting for the pDJDC plan is also shown (b).

In pDJDC plans, we used a beam set with 2–7 ports to targets at the same level in the craniocaudal direction, and employed another beam set for other targets using different port angles (9–12 angles in total). The couch moves rapidly during the intervals between the different beam sets (Fig. 2). Figure 3 shows the typical case (B in Table 1). This patient had multiple bone metastases including ribs, vertebrae, and the pelvis [Fig. 3(a)]. In the pDJDC plan, two oblique fields were set for the rib and another 7‐port beam set was employed for the other targets [Fig. 3(b)]. Figure 4 shows the similar case (G in Table 1). The targets were separated into three groups according to the level in the craniocaudal direction, and a four‐port beam set was employed for each group [Fig. 4(b)]. In HDJ plans, artificial blocks were necessary; these were used as a “complete block” in the optimization procedure, to avoid the lung and liver when the targets were in the rib or scapula [Figs. 3(d), 4(d)]. A 5.0‐cm dynamic‐jaw, large pitch (0.430–0.500), and small modulation factor (1.1–2.0) were used for all plans to reduce beam‐on time. However, when the calculated gantry period (time for one gantry rotation) was more than 60 sec in the HDJ plans, a smaller pitch had to be used to reduce the gantry period due to the limitations of the system.

The total doses and fractionations for each patient are summarized in Table 1. The doses were prescribed for the PTVs as median doses.^14^ The inverse planning procedure of optimization using the tomotherapy planning station was described in detail previously.^15^ A fine calculation grid (1.95 × 1.95 mm) was used for the final calculation process.

### 2.C Plan Evaluation

To compare the pDJDC and HDJ plans, the conformity index, uniformity index, and beam‐on time were evaluated in the tomotherapy planning system. The conformity index and uniformity index were calculated according to the following formulae:^15^, ^16^

$$\text{Conformity}\;\text{index}=\left(V_{\text{PTV}}/{\text{TV}}_{\text{PV}}\right)/\left({\text{TV}}_{\text{PV}}/V_{\text{TV}}\right)$$

Uniformityindex=D5%/D95%

where V_PTV_ = PTV (ml), TV_PV_ = lesion volume (ml) covered by the prescribed isodose, V_TV_ = prescribed isodose volume (ml), and D5% = minimum dose delivered to 5% of the PTV. A lower conformity index indicates higher conformity, and a lower uniformity index indicates better homogeneity. The ideal conformity index and uniformity index are both 1.

For five patients with thoracic targets, dose‐distribution parameters in the lung (V5Gy, V20Gy, mean lung dose) were also evaluated. VxGy represents the percentage or absolute volume (V) receiving the specified dose (x) in Gy, for example, V5Gy is the percentage volume receiving 5 Gy.

### 2.D Treatment time

The beam‐on time was calculated automatically, but it did not include the interval time for gantry rotation and couch travel between ports in the pDJDC plans. During the intervals, the couch moves much faster (75 mm/sec) than during the beam‐on time. Couch traveling lengths at the intervals were within 375 mm (5 sec) in almost all treatments in this study. The gantry rotation speed is 36 degree/sec during the intervals. Thus, the longest gantry traveling time during the intervals is calculated to be 5 sec (moving 180 degrees). We measured the interval time when treating the patients in this study with the pDJDC technique, and confirmed that each interval was 5 sec or less. Based on this calculation and observation, we calculated the treatment times as follows:

$$\text{pDJDC}\;\text{plans}:\text{treatment}\;\text{time}\;=\;\text{beam}-\text{on}\;\text{time}+\left(\text{number}\;\text{of}\;\text{ports}-1\right)\times 5\;\text{seconds}$$

HDJplans:treatmenttime=beam-ontimeincludingcouchtraveltime

### 2.E Statistical analysis

The conformity index, uniformity index, dose distribution in the lung, monitor unit, and beam‐on time were compared using the Wilcoxon signed‐rank test. Statistical analyses were carried out with the statistical software package “R.”^17^ All planning and evaluation was performed by one radiation oncologist (first author).

## 3 RESULTS

**Fig. 5:**
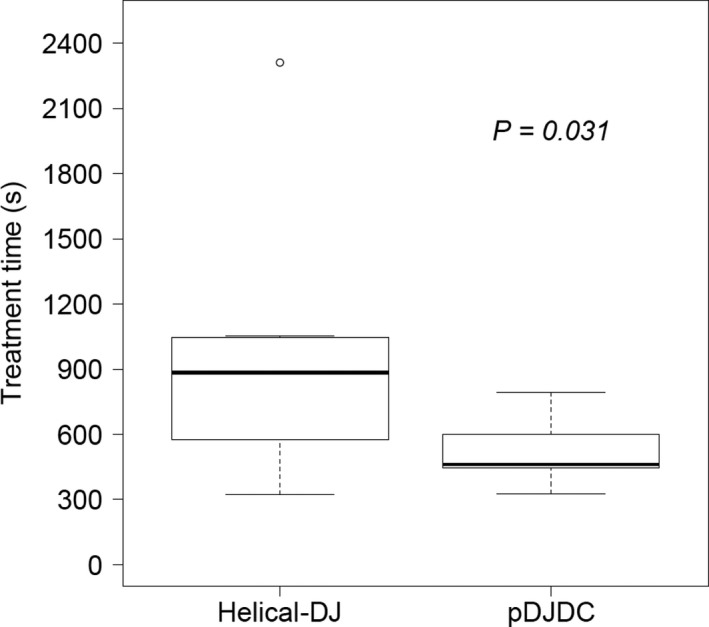
Treatment times of the plans for seven patients with multiple targets using helical tomotherapy with dynamic‐jaw mode (Helical‐DJ) and the pseudo‐dynamic‐jaw and dynamic‐couch technique (pDJDC).

Figures 3 and 4 show the representative dose distributions for the two plans in a patient with multiple bone metastases. The dose distribution appeared to be better in the HDJ plans because of the better conformity, but the treatment time was much longer than in the pDJDC plans. The treatment parameters, dose–volume parameters, beam‐on time, treatment time, and monitor units of the two plans for the seven patients are summarized in Table 2. The median conformity index deteriorated in the pDJDC plans, but the uniformity index and the dose distribution of the lung were similar. The pDJDC plans reduced the treatment time by 48% compared to the HDJ plans (median: 462 and 884 sec, respectively, *P* = 0.031, Fig. 5).

**Table 2:** Treatment and dose–volume parameters, monitor units, beam‐on times, and treatment times of the two plans.

Median (range)			
	pDJDC	HDJ	*P* ^a^
Patient number	7		
Pitch	0.500 (0.500–0.500)	0.430 (0.215–0.500)	0.031
Modulation factor	1.70 (1.10–1.81)	2.34 (1.80–4.25)	0.016
Monitor unit	5351 (2733–8690)	13600 (4443–32800)	0.016
Beam‐on time	422 (287–736)	884 (322–2312)	0.016
Treatment time^b^	462 (327–795)	884 (322–2312)	0.031
Conformity index	2.98 (2.24–9.70)	2.44 (1.94–6.89)	0.031
Uniformity index	1.05 (1.02–1.50)	1.06 (1.01–1.21)	0.10
Lung^c^			
V5Gy (%)	11.8 (3.32–21.7)	8.48 (7.19–15.5)	0.63
V20Gy (%)	1.62 (0.23–5.46)	2.13(0.24–4.89)	0.31
Mean lung dose	2.62 (1.35–3.47)	2.35 (1.83–3.42)	0.63

Abbreviations: pDJDC = pseudo‐dynamic‐jaw and dynamic‐couch technique, HDJ = helical‐dynamic‐jaw mode.

^a^ *P* value between pDJDC and HDJ plans.

^b^ pDJDC plans: treatment time = beam‐on time + (number of ports – 1) × 5 sec HDJ plans: treatment time = beam‐on time including couch travel time

^c^ For five patients with thoracic targets.

## 4 DISCUSSION

This study revealed that the pDJDC technique allows tomotherapy treatment of multiple targets in about half the time required for the HDJ technique. This technique may be used for any sites in the body, but is especially useful for patients with multiple targets including thoracic lesions; using oblique static ports, the lungs can be avoided easily without using artificial lung blocks and by increasing the modulation factor. We previously reported the usefulness of static‐port tomotherapy for thoracic tumors.^10^, ^11^, ^15^

In HDJ plans, an artificial complete block is necessary to avoid the lungs when some of the targets are located in the thorax, and this results in a longer gantry period. For example, to treat the rib tumor in the patient in Fig. 3, the beam‐on gantry angle was extremely limited due to the complete block. Thus, the gantry would move slowly to deliver the dose to the target from limited angles. The gantry rotates at a uniform pace throughout the session, resulting in a long treatment time. When the dose of a fraction is high, the gantry periods can be more than 60 sec, and thus the plan will not be able to be implemented in practice due to limitations of the system. To avoid this limitation of the gantry period, a smaller pitch and more rotations would be needed to cover all the targets, extending the treatment time even further. Thus, when some of the targets exist in the thoracic region, this technique may be useful even when the distance between the targets is not so large.

The median conformity index deteriorated in the pDJDC plans due to the limited number of available ports. However, patients with multiple targets often suffer from pain, and treatment time is an important issue for those patients. The reduction of treatment time will contribute not only to relief of stress for them but also to improvement of positional accuracy. The maximum number of ports is 12, and the targets must be separately located in the craniocaudal direction to use the pDJDC technique; we should thus attempt to cover the targets within 2–9 fields for a group of targets. Using more ports will increase the conformity, but the beam‐on time will also increase. The balance of conformity and beam‐on time should be taken into consideration. From this point of view, the technique is suitable for patients with multiple targets that are readily divided into 2–3 groups in the craniocaudal direction. If the targets are divided into more than three groups in the craniocaudal direction, only 2–3 beams can be used for each group and the conformity deteriorates. On the other hand, when all the targets are located at a same level, this technique cannot be used.

In HDJ treatment, beams are generated continuously even during the gantry is passing between the targets. At that time, all leaves of the multi‐leaf collimator are closed, but the jaw is open with 1‐cm width. Thus, patients receive leakage radiation. In pDJDC treatment, the beams are off between the targets. This should be another advantage of the pDJDC technique.

As a limitation of this study, the patient number was small. Especially, the lung dose could only be evaluated for five patients harboring thoracic targets. We will continue to evaluate this technique for more patients with an indication stated above.

In the future, helical‐dynamic‐jaw and dynamic‐couch modes may become available for clinical use. In our opinion, however, this pDJDC technique will still be useful when some of the targets are in the thoracic region.

## 5 CONCLUSION

The pDJDC technique allows treatment of multiple targets in almost half the time of the HDJ technique. The pDJDC plans were comparable to the HDJ plans in dose distribution, but the conformity index deteriorated.

## CONFLICT OF INTEREST

The authors declare that they have no conflicts of interest.
